# A case report of shingles and subsequent cardiac ischaemia in a young male

**DOI:** 10.1093/ehjcr/ytag473

**Published:** 2026-06-24

**Authors:** Katelyn Eisendrath, Shanna Horne, Lindsay Edmondson, Andrew West, Dakota Robertson

**Affiliations:** Department of Internal Medicine, Johnston Memorial Hospital, 16000 Johnston Memorial Drive, Abingdon, VA 24211, USA; Department of Internal Medicine, Johnston Memorial Hospital, 16000 Johnston Memorial Drive, Abingdon, VA 24211, USA; Department of Internal Medicine, Johnston Memorial Hospital, 16000 Johnston Memorial Drive, Abingdon, VA 24211, USA; Department of Interventional Cardiology, Johnston Memorial Hospital, 16000 Johnston Memorial Drive, Abingdon, VA 24211, USA; Department of Internal Medicine, Johnston Memorial Hospital, 16000 Johnston Memorial Drive, Abingdon, VA 24211, USA

**Keywords:** Cardiac ischemia, NSTEMI, Shingles

## Abstract

**Background:**

A type 1 non-ST-segment elevation myocardial infarction (NSTEMI) is a rare complication of shingles infection, especially in young individuals with few predisposing risk factors.

**Case summary:**

A 39-year-old man, who was successfully treated for herpes zoster meningitis and encephalitis 3 months before this hospitalization, presented to the emergency department with worsening chest pain that radiated to his back with associated shortness of breath. Based on elevated troponin and abnormalities seen on electrocardiogram and transthoracic echocardiogram, the patient was diagnosed with a type 1 NSTEMI. A left heart catheterization was performed, resulting in a drug-eluting stent to the left anterior descending ostium. He was discharged with oral aspirin 81 mg daily, oral ticagrelor 90 mg twice daily, and oral rosuvastatin 20 mg daily.

**Conclusions:**

Infection with herpes zoster virus (HZV) has been temporally associated with increased risk of cardiovascular disease, even in those who are young with few predisposing risk factors. It has been postulated that HZV infection causes inflammation, subsequent hypercoagulability, vessel ischaemia, and arterial remodelling. More research needs to be conducted to further understand the possible mechanisms of action of HZV infection’s effect on the heart.

Learning points

- Age and time after herpes zoster infection are inversely correlated to cardiovascular complications, including cardiac ischaemia.
- It has been postulated that herpes zoster virus infection contributes to the development of inflammation, subsequent hypercoagulability, vessel ischaemia, and arterial remodelling.

## Introduction

According to the Centers for Disease Control and Prevention, one in three individuals are infected with shingles, also known as herpes zoster virus (HZV), at least once in their lifetime.^1^ About one million people are diagnosed with HZV every year, and the incidence of shingles infection correlates positively with increasing age.^1,2^ Complications of this infection can include the development of postherpetic neuralgia, coronary artery disease, and stroke.^1,2^ Recent studies have noted an increase in the risk of cardiovascular and cerebrovascular events within one year following HZV infection. This has been attributed to multiple factors, including inflammatory responses, remodelling, and vasculopathy.^3^ This case highlights the occurrence of cardiac ischaemia within three months of prior HZV infection in a young male with limited predisposing risk factors.

## Summary figure

**Figure:**
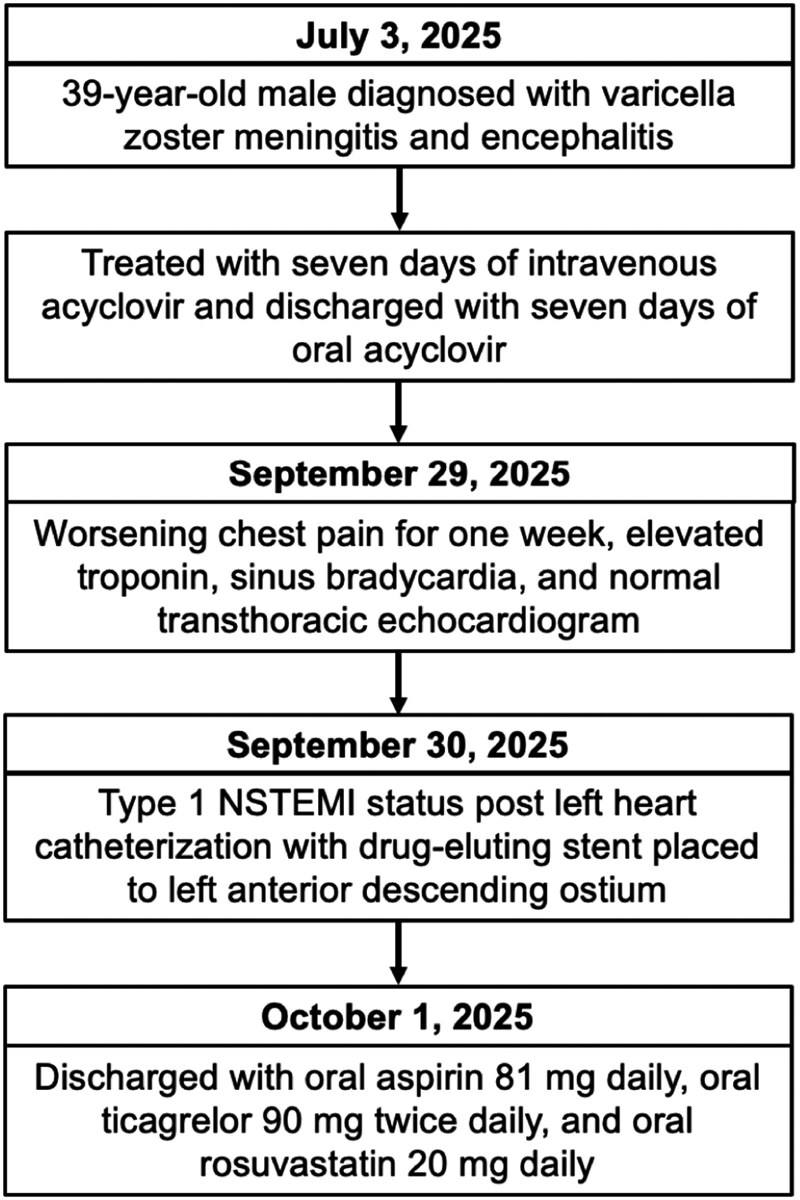


## Case presentation

A 39-year-old male presented to the emergency department on 29 September 2025, with chief complaints of chest pain and shortness of breath. His medical history included a shingles infection complicated by varicella zoster meningitis, encephalitis, and postherpetic neuralgia diagnosed on 3 July 2025. He was successfully treated with seven days of intravenous acyclovir followed by seven days of oral acyclovir 800 mg twice daily.

At the time of presentation, the patient described his chest pain as a pressure-like sensation that radiated to his back with associated shortness of breath. His chest pain had been worsening over the course of a week. It initially occurred with activity and then progressed to occurring at rest. He was a physically active young male with no prior history of smoking, hypertension, type 2 diabetes mellitus, or family history of premature coronary artery disease.

Pertinent lab work included hyperlipidaemia and elevated troponin with a body mass index (BMI) of 31. His lipid profile at presentation was as follows: cholesterol 234 mg/dl, triglycerides 294 mg/dl, HDL 30 mg/dl, and LDL 145 mg/dl. His elevated troponin peaked at 0.36 ng/ml (normal < 0.04 ng/ml) but trended as follows: 0.34 ng/ml, 0.36 ng/ml, 0.33 ng/ml, 0.35 ng/ml, 0.34 ng/ml, 0.21 ng/ml, 0.16 ng/ml. Electrocardiogram revealed sinus bradycardia and Q waves in leads V1/V2. Transthoracic echocardiography revealed left ventricular ejection fraction of about 65%, normal diastolic function, and mild concentric left ventricular hypertrophy. He was diagnosed with a type 1 non-ST-segment elevation myocardial infarction (NSTEMI) due to elevated troponin and persistent typical chest pain, which raised concern for plaque rupture.

The patient then underwent cardiac catheterization on 30 September 2026, which revealed abnormal pathology. The left anterior descending (LAD) artery was a very large calibre vessel that immediately tapered into a tubular segment of 90% narrowing just inside the ostium, with thrombolysis in myocardial infarction (TIMI) 2 antegrade flow present. The circumflex artery was a large calibre vessel with 25% diffuse irregular narrowing distally. The right coronary artery was a moderate-calibre vessel with 40% tubular narrowing in the middle third and a focal segment of 50% narrowing in the terminal portion. Percutaneous coronary intervention included a 4 × 15 Xience drug-eluting stent placed just inside the LAD ostium with post-intervention TIMI flow of 3. Intravascular ultrasound demonstrated good stent deployment and appeared well opposed. The patient tolerated the procedure well and was discharged on oral aspirin 81 mg daily, oral ticagrelor 90 mg twice daily, and oral rosuvastatin 20 mg daily. The patient was seen in the outpatient clinic 1 month later and reported no further cardiac symptoms. A follow-up exercise stress test revealed excellent exercise capacity with normal myocardial perfusion.

This interesting case draws attention to the possible relationship between cardiac ischaemia and HZV infection, as well as the pathophysiological mechanism that connects the two.

## Discussion

Varicella zoster virus (VZV), which can later be reactivated as HZV, affects many individuals worldwide and can have life-vs.-death consequences in some cases. Initial infection with VZV, also known as chickenpox, often occurs during childhood.^4,5^ While chickenpox usually resolves with symptomatic treatment over the course of seven to ten days, the virus lies dormant in the dorsal root ganglia.^4,5^ When reactivated at a later time, HZV develops and causes severe shock-like pain in a dermatomal distribution for an average of forty-five days.^4,5^ Not only does HZV cause this pain, but it has also been found to be temporally associated with increased risk of cardiac ischaemia and stroke, especially in younger ages and within one year of infection.

A comprehensive review revealed that age and time after HZV infection negatively correlate with cardiovascular complications.^3^ Thus, the younger a patient presents with shingles infection, the higher the likelihood that this individual is to have a cardiac event. Furthermore, another study found that the risk of a major cardiovascular event increases by about 30% in those who have had a prior HZV infection, and this risk can continue for more than 12 years.^6^ However, the risk is highest in the first month following VZV diagnosis.^7^

The pathophysiological mechanism as to how HZV is potentially associated with increased incidence of coronary artery disease has yet to be established. There are multiple ways by which HZV is suspected to inflict damage to vessel walls, including inflammatory responses, remodelling, and vasculopathy.^3,8–10^ Ongoing research has postulated that HZV infection contributes to inflammation and subsequent hypercoagulability and vessel ischaemia.^8,9^ The virus itself stimulates prothrombotic antibodies, including IgM and IgG anticardiolipin, and the human immune system generates immune complexes, both of which contribute to vascular remodelling.^3^ Furthermore, vasculopathy can be associated with viral infections, creating subsequent inflammation of the vessel wall.^10^ It has been demonstrated in affected arterial walls showing internal elastic lamina disruption, hyperplasia of the intima, and a reduced amount of smooth muscle cells.^10^ Collectively, these processes cause endothelial dysfunction and contribute to the destabilization of existing atherosclerotic plaque. This is consistent with the increased risk seen in those with pre-existing cardiovascular risk factors.^3^

In our case, the patient did have an elevated BMI and dyslipidaemia, but he otherwise had no additional predisposing risk factors. He had no prior history of smoking, hypertension, type 2 diabetes mellitus, or family history of premature coronary artery disease. It is possible that our patient suffered from premature atherosclerotic coronary disease with subsequent HZV-driven inflammatory plaque destabilization. It can be speculated that HZV-related vascular inflammation contributed to this patient’s case due to his young age and relatively short time between being diagnosed with shingles complicated by varicella zoster meningitis and the type 1 NSTEMI. More research should be conducted to further understand the exact mechanisms by which HZV affects the cardiovascular system.

Furthermore, this case report is particularly interesting due to the young age of the patient. Most individuals who develop HZV infection are diagnosed after age 50, which is why two doses of the recombinant zoster vaccine are recommended after age 50.^11^ It can be surmised that at the age of 39, this patient was not vaccinated against HZV, which made him more susceptible to a subsequent cardiac event. Additional future research should focus on the recombinant zoster vaccine’s preventative vascular effects of shingles infection.

## Limitations

Although temporal association and biologic plausibility exist, definitive causality between HZV infection and myocardial ischaemia cannot be established from a single observational case. Furthermore, the absence of intracoronary imaging limits the precise characterization of the lesion substrate and differentiation between inflammatory vasculopathy and premature atherosclerotic disease.

## Data Availability

No new data were generated or analysed in support of this research.

## Lead author biography

Katelyn Eisendrath, DO, MS, is an Internal Medicine Resident Physician at Johnston Memorial Hospital. She will be applying to cardiovascular disease fellowships in the near future. Dr. Eisendrath attained her medical degree at Kansas City University College of Osteopathic Medicine, a Master of Science in Physiology degree at the University of Michigan, and a Bachelor of Science degree at the University of Michigan.

## Notes

**Consent:** The patient provided written consent for this case to be published in a journal, in compliance with COPE guidelines.
